# Physical and Antibacterial Properties of Sodium Alginate—Sodium Carboxymethylcellulose Films Containing *Lactococcus lactis*

**DOI:** 10.3390/molecules23102645

**Published:** 2018-10-15

**Authors:** Jingsong Ye, Donghui Ma, Wen Qin, Yaowen Liu

**Affiliations:** 1 College of Food Science, Sichuan Agricultural University, Ya’an 625014, China; yjsh529@yahoo.com.cn (J.Y.); 18894314357@163.com (D.M.); qinwen@sicau.edu.cn (W.Q.); 2 School of Materials Science and Engineering, Southwest Jiaotong University, Chengdu 610031, China

**Keywords:** edible films, *Lactococcus lactis*, sodium alginate, sodium carboxymethylcellulose

## Abstract

Edible films have gradually become a research focus for food packaging materials due to a variety of benefits, including environmental friendliness, good barrier properties, and good carrying capacity. In this experimental study, we used sodium alginate as a film-forming substrate, sodium carboxymethylcellulose as a modifier, and glycerol as a plasticizer, then *Lactococcus lactis* was added to film solutions to form bacteriostatic films via the tape casting method. With the addition of *Lactococcus lactis*, the films did not significantly change thickness, while the transparency decreased and a significant increase in red and yellow hues was observed. Meanwhile, the dispersion of bacterial cells in film solutions destroyed intermolecular interactions in the solutions during film formation and increased the volume of voids in the *Lactococcus lactis*-containing films, thereby slightly decreasing the tensile strength of the films, but significantly increasing water vapor permeability. Moreover, the films with added *Lactococcus lactis* showed significant bacteriostatic activity against *Staphylococcus aureus* at 4 °C. In a seven-day bacteriostatic test, the films with *Lactococcus lactis* added at a level of 1.5 g/100 g resulted in a decrease in the viable cell count of *Staphylococcus aureus* by at least four logarithmic units. This study of *Lactococcus lactis*-containing films has provided a new method and strategy for antibacterial preservation of foods.

## 1. Introduction

The Food and Agriculture Organization of the United Nations and the World Health Organization (WHO) define probiotics as active beneficial microorganisms that colonize the human gut and reproductive system and can provide definite health benefits, thereby playing a beneficial role in improving the microecological balance of the host [1]. Studies have shown that the intake of a sufficient number of probiotics is beneficial to the health of the host, including maintaining intestinal homeostasis [2], fighting pathogenic intestinal bacteria, rebuilding intestinal flora [3], regulating the intestinal immune system, suppressing the damage of toxins in the intestine, and ensuring intestinal nutrient metabolism [4]. Lactic acid bacteria act as probiotics in the human body and are collectively a group of nonsporulating, Gram-positive bacteria that can produce large amounts of lactic acid in the fermentation of carbohydrates. They can regulate microbial flora in the organism, maintain microecological balance, inhibit the activity of pathogenic bacteria in the digestive tract and reduce their adhesion to the intestinal lining, and restore the physiological function of the intestines [5]. Lactic acid bacteria can enhance the barrier function of epithelial cells by stimulating intestinal epithelial cells to secrete mucin, which in turn reduces the risk of pathogenic bacterial infection [6]. Lactic acid bacteria can also inhibit pathogenic bacteria in the intestinal tract by producing lactic acid and bacteriocin. Moreover, lactic acid bacteria have been widely used in food preservation since ancient times, as these bacteria and their metabolites (organic acids, hydrogen peroxide, carbon dioxide, and bacteriocin) are effective for bacteriostatic preservation [7]. Studies have shown that lactic acid bacteria have a good bacteriostatic preservation effect, but as they metabolize carbohydrates for growth, their direct application to fresh foods is likely to cause the total number of bacterial colonies to exceed standards, which greatly limits their use in this application [8]. Therefore, it is essential to explore appropriate methods for taking advantage of the bacteriostatic preservation effect while limiting the number of bacterial colonies on fresh foods from exceeding the standard; this has motivated the development of edible films [9].

The most commonly used method for preparing edible films is based on microencapsulation technology [10], in which functional components are first coated in a mesh-like network formed by natural polymers, then slowly released during application [11]. For lactic acid bacteria-containing edible films, active lactic acid bacteria are embedded in films to achieve the bacteriostatic preservation effect of the bacteria [12]. Gialamas et al. used sodium caseinate as a film-forming substrate to prepare *Lactobacillus sakei*-containing films by directly spraying the bacteria onto the films or by embedding them in the films. They compared the two film preparation methods for changes in the viable-cell count of *Lactobacillus sakei* in the films stored at 4 °C or 25 °C and found that within 30 days of storage, the embedding method had a significantly better effect than the spraying method [13]. Sánchez-González et al. used cellulose, methylcellulose, soy protein, and sodium caseinate as film-forming substrates to study the cell viability of *Lactobacillus plantarum* after film formation and found that the cell viability was lower in cellulose edible films than in edible protein films. The study also showed that *Lactobacillus plantarum*-containing cellulose/protein edible films could suppress Listeria monocytogenes and showed good antibacterial efficacy [14]. Since the number of live lactic acid bacteria decreased during storage, Soukoulis et al. used gelatin as a film-forming substrate in an attempt to increase the cell viability of lactic acid bacteria by adding prebiotics, including inulin, dextran, oligodextran, and maltose. They found that the addition of the prebiotics increased the cell viability of lactic acid bacteria in the edible films during storage [15]. The lactic acid bacteria-containing pullulan/starch edible films prepared by Kanmani et al. showed a cell viability of 70–80% for three species of lactic acid bacteria after two months of storage at 4 °C, proving that the pullulan/starch mixture was a good carrier for preparing lactic acid bacteria-containing edible films [16]. Currently, there are few studies regarding the development of synthetic films incorporating bacteria [17], The *Lactococcus lactis subsp. lactis*-containing polyvinyl alcohol (PVOH) synthetic films prepared by Laura Settier-Ramírez et al. showed a cell viability of *L. lactis* in the films developed stored at 20 °C and 43.2% relative humidity for four weeks, and the antimicrobial activity against *L. monocytogenes* were determined [18].

Due to their good moisture retention, micro-organism resistance, and air permeability, lactic acid bacteria-containing edible films have recently been widely used in food preservation to inhibit food browning [19], reduce nutrient loss, and prolong the period of food preservation [20]. Soukoulis et al. investigated the effect of a *Lactobacillus rhamnosus* coating on bread quality and found that the viable cell count of *Lactobacillus rhamnosus* decreased significantly within 24 h of bread storage and then continued to slightly decrease for 2–3 days and then began to increase within 4–7 days of storage. A simulated digestion test showed that the viable cell counts of *Lactobacillus rhamnosus* before and after digestion were 7.57–8.98 and 6.55–6.91 log (CFU/bread slice), respectively, satisfying the WHO standard for viable cell counts of lactic acid bacteria in functional foods, providing a new strategy for the development of foods containing live lactic acid bacteria [15]. Tavera-Quiroz et al. studied the preservation effect of a *Lactobacillus plantarum*-methylcellulose coating on green apple snacks and found that the viable cell count of *Lactococcus lactis* after 90 d of storage at 20 °C under 60% relative humidity was (2.0 ± 0.7) × 10^8^ CFU/g, a value far above the minimum required number of 10^6^–10^7^ CFU/g for probiotics. Later, a storage experiment of green apple snacks showed that the total microbial count on the snack surface decreased by 1.4 log CFU/g, suggesting that the *Lactobacillus plantarum*-methylcellulose coating had a certain bacteriostatic preservation effect [21].

In this study, we propose a new idea for the antimicrobial storage of foods using sodium alginate (SA) as a film-forming substrate, sodium carboxymethylcellulose (CMC) as a modifier, and glycerol as a plasticizer to prepare *Lactococcus lactis (Lla)*-containing films with a bacteriostatic preservation effect.

## 2. Discussion and Results

### 2.1. Survival of Lactococcus Lactis Strains

**Figure 1:**
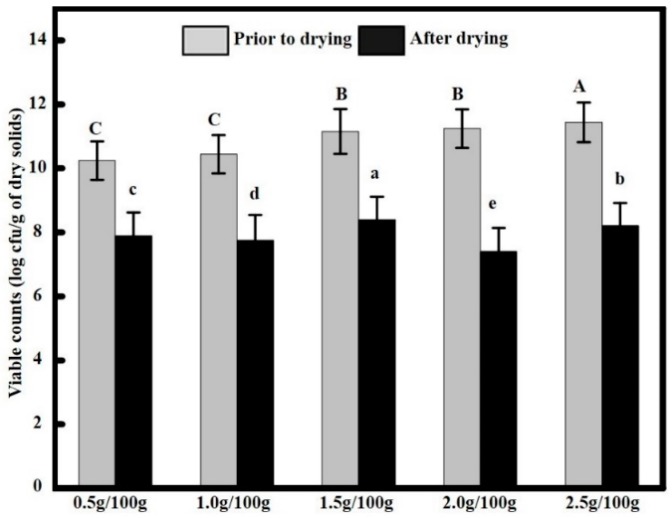
Survival of *Lactococcus lactis* throughout air drying at 30 °C for 48 h. Data followed by different capital letter are significantly different (*p* < 0.05) when comparing viable counts prior to drying. Data followed by different lowercase letters are significantly different (*p* < 0.05) when comparing viable counts after drying.

We observed that not all *Lactococcus lactis* survived the film drying process. Viable cell counts of *Lactococcus lactis* before and after film drying were measured with a serial dilution method, and the results are shown in Figure 1. When the added bacteria content increased from 0.5 g/100 g up to 2.5 g/100 g of film solution, the viable cell counts prior to film drying were 10.24 ± 0.88, 10.44 ± 0.91, 11.14 ± 0.96, 11.24 ± 1.02, and 11.44 ± 1.08 log CFU/cm^2^, respectively, while after 48 h of drying at 30 °C, the viable cell counts in the edible films decreased to 7.89 ± 0.61, 7.75 ± 0.58, 8.39 ± 0.70, 7.40 ± 0.56, and 8.20 ± 0.68 log CFU/g, respectively. The pH of the film without *Lactococcus lactis* was 6.5 ± 0.2, but we observed that the pH of the films containing *Lactococcus lactis* decreased. When the added bacteria content increased from 0.5 g/100 g up to 2.5 g/100 g of film solution, the pH of the films decreased to 6.4 ± 0.4, 6.4 ±0.3, 6.4 ± 0.3, 6.3 ± 0.3, 6.2 ± 0.2, while the *Lactococcus lactis* content had no significant effect on the pH of the edible films (*p* > 0.05). We found a counterintuitive, yet very interesting, phenomenon in contrast to the monotonic increase in the concentration of the initial bacterial solutions; the viable cell counts after drying did not increase in a positively correlated, monotonic manner, where the 1.5 g/100 g group showed the largest viable cell count. Given that the viability of living cells in a heating process is affected by multiple factors [22], it is speculated in this study that the viability of *Lactococcus lactis* in the film solutions should not be interpreted solely from the added bacteria content, but from a holistic perspective of the edible film composition [23], water activity [16], and fluid osmotic pressure [24], as well as the environmental pH. Moreover, it was found that our experimental results were in agreement with those of Sánchez-González et al. [14].

### 2.2. Morphology of the Films

**Figure 2:**
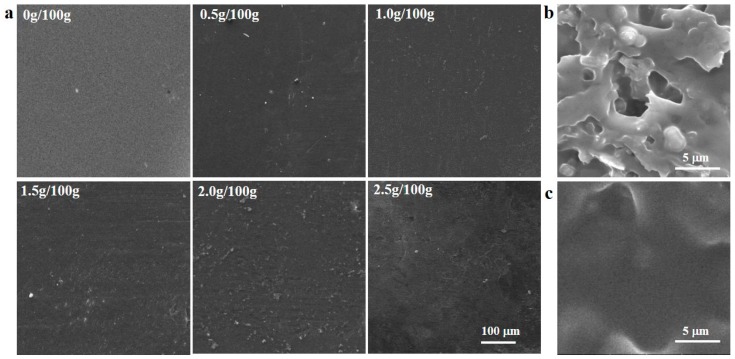
(**a**) Scanning electron microscope (SEM) micrographs of the films added with different concentrations (g/100 g) of *Lactococcus lactis*; (**b**) SEM of the cross-section of the films added with 1.5 g/100 g *Lactococcus lactis*; (**c**) SEM of the cross-section of the films added with 0 g/100 g *Lactococcus lactis*.

Figure 2a shows scanning electron microscope (SEM) images of the surface morphology of the *Lactococcus lactis*-containing sodium alginate-sodium carboxymethylcellulose edible films. As shown in the left panels of the two figures, the edible films incorporated with 0.5 g/100 g, 1.0 g/100 g, and 1.5 g/100 g of *Lactococcus lactis* had smooth surfaces with no cracks, indicating that these compositions resulted in good compatibility between the components, indicating that the added contents of *Lactococcus lactis* were appropriate and the method was reliable [15]. In contrast, the films containing 2.0 g/100 g and 2.5 g/100 g of *Lactococcus lactis* showed aggregation of the bacteria and poor distribution over the film area. This indicated that the bacteria were poorly incorporated in the films, leading to bacterial pairing or chaining and irregular edges; as it has been also confirmed in previous studies [25]. The addition of *Lactococcus lactis* resulted in detectable changes in the microstructure of the films (Figure 2b). As illustrated in the SEM of cross-section micrographs from control and *Lactococcus lactis*-containing film, the structure of control film was homogeneous, uniform and compact with no micropores (Figure 2c). However, *Lactococcus lactis*-containing film showed a higher number of holes than control films. The *Lactococcus lactis* were still spherical or ovoid in shape, had a smooth surface, and were evenly embedded in the edible films, that could result in increasing *Lactococcus lactis*-protective effects of films. These results were also confirmed by increasing the water vapor permeability (WVP) of *Lactococcus lactis*-containing films and decreasing their mechanical property (Table 1). This is in contrary to the findings of Odila Pereira et al. who reported that the effect of microorganism incorporation on the structural conformation of films was insignificant [26].

### 2.3. Optical Properties of Films

The optical properties, including the color and transparency of the films, were analyzed, as these characteristics directly affect the appearance of packaged products. With increasing content of bacterial cells, the color of the films changed, as shown in Table 2. In general, the *Lactococcus lactis* content had a significant effect on the color of the edible films (*p* < 0.05). It was found that the L* value of the films decreased from 89.81 ± 1.81 to 87.06 ± 1.28, while the b* values tended to increase, from 3.52 ± 0.35 to 7.69 ± 0.95, indicating that the film transparency decreased to some extent; similar observations were also made by us from the research results of other researchers [27]. The film luster is usually related to the gloss. What is more, the luster of the films is related to their surface morphology after drying, with a smoother surface giving a higher degree of luster [28]. This is primarily attributed to the microbial cells on the surface of the films having a different density than the polymer solutions, resulting in a continuous, irregular layer being formed during drying. This results in a rough film surface that increases the optical refraction and decreases film transparency, and changes the surface color. In addition, the microbial cells fixed in a region near the film surface became isolated and changed the refractive index of this region, affecting the surface optical properties [14]. Moreover, according to the research of Ly et al., the effect of bacteria on the interfacial properties of other compounds is mainly attributed to the surface charge of the bacteria, which determines the electrostatic interaction between the bacteria and charged polymers, and in turn, determines the film luster (which is irrelevant to cell viability) [29].

### 2.4. Mechanical Properties of Films

Given that the edible films are the matrixes for *Lactococcus lactis* and the main body of the food package, the tensile strength (TS) and elongation (E) at break are two key indicators of the applicability of edible films. Edible films should be able to provide a stable environment to ensure normal viability of the *Lactococcus lactis*, while maintaining sufficient plasticity to avoid package rupture during food packaging and storage [9]. With the addition of bacteria, the film thickness increased, while the *Lactococcus lactis* content had no significant effect on the thickness of the edible films (*p* > 0.05) (Table 1), which agreed with the finding of Brachkova et al. [30]. In contrast, the addition of *Lactococcus lactis* had an adverse effect on the TS and E of the *Lactococcus lactis*-containing edible films, where these two values showed a significant (*p* < 0.05) downward trend, where TS decreased from the initial value of 27.23 ± 3.19 MPa to a final value of 7.50 ± 1.01 MPa and E% decreased from 24.86 ± 1.65% to 9.19 ± 1.03%. In particular, when the bacteria content increased from 0.5 g/100 g to 1.5 g/100 g of film solution, the mechanical properties of the films did not decrease significantly; this could be attributed to the increased film thickness. When the bacteria content exceeded 2 g, the bacterial cells were dispersed in the film solutions; during film formation, the cells interrupted inter-macromolecular interactions in the film solutions, which in turn decreased the cohesive force in the polymer network, decreasing the TS of the *Lactococcus lactis*-containing edible films. With further addition of the bacteria, this effect became more obvious, leading to a more significant decrease in TS and E%. Maybe the addition of the bacteria destroyed the biomacromolecule interlinking, the hydrogen bonding force between sodium alginate and sodium carboxymethylcellulose, and the intramolecular hydrogen bonding force were weakened, resulting in reduced TS [16].

### 2.5. Water Vapor Permeability

The WVP of a film is one of the most important parameters to evaluate the feasibility of the film as a packaging material, as this property could affect the ability of foods to exchange moisture with the environment. The effect of the *Lactococcus lactis* content on the WVP of the edible films is shown in Table 1. The WVP increased with increasing bacteria content, from an initial value of 6.73 × 10^−11^ gm/m^2^ s Pa to a final value of 10.25 × 10^−11^ gm/m^2^ s Pa, where similar phenomena were previously reported in many studies [31]. This behavior was attributed to two factors: Firstly, the evaporation of water during drying can lead to the formation of films of high continuity and compactness; secondly, sodium alginate and sodium carboxymethylcellulose contain hydrophilic -OH groups. When the bacteria content increased from 0 g/100 g to 1.5 g/100 g of film solution, WVP increased from 6.73 ± 0.86 × 10^−11^ gm/m^2^ s Pa to 10.25 ± 1.25 × 10^−11^ gm/m^2^ s Pa. Although the WVP values generally showed an increasing trend, there was no significant difference in the values (*p* > 0.05). Although the film thickness will affect the WVP, it was not the main factor contributing to the observed changes in WVP [13]. When the bacteria content was higher than 2 g/100 g, the WVP of the films showed a dramatic increase (*p* < 0.05). It was suspected in this study that the *Lactococcus lactis* content affected the WVP of the films; that is, the addition of a large quantity of *Lactococcus lactis* changed the spatial structure of the molecules and increased the intermolecular space. Soukoulis et al. also reported that after dispersing bacterial cells into film solutions, the inter-macromolecular interactions were destroyed during film formation so that the voids in the films were enlarged, allowing water vapor to pass through the edible films at higher rates, which directly increased the WVP [32].

### 2.6. Film Moisture Content

**Figure 3:**
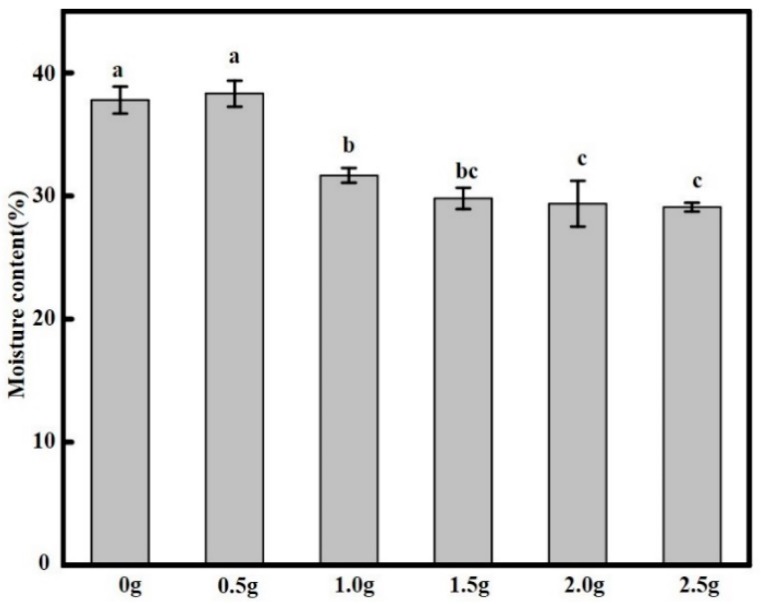
Moisture content of SA/CMC films added with different concentrations (g/100 g) of *Lactococcus lactis*. Data followed by different lowercase letters are significantly different (*p* < 0.05) when comparing moisture content.

The moisture content (MC) affects the long-term cell viability of *Lactococcus lactis* in the films, and it also has a certain effect on the film solubility [16]. Therefore, it is highly necessary to determine the MC of edible films, as shown in Figure 3. Our experiments revealed that the MC in the films was between 29.09 ± 2.67% to 38.30 ± 3.14%, where the addition of *Lactococcus lactis* had significant effect on the moisture content (*p* < 0.05). Moreover, it was observed that low *Lactococcus lactis* contents increased the water content of the films, a phenomenon similar with that observed by Ebrahimi et al. [33], which may be attributed to the dehydration of SA/CMC during film formation. The relative content of hydrophilic substances in the films decreased with increasing bacteria content, certainly related to the existence of possible interactions between *Lactococcus lactis* and SA/CMC, causing a decrease MC of the films. In addition, as the number of *Lactococcus lactis* increased, the film thickness increased, which in turn reduced water retention in the films [27].

### 2.7. Fourier Transform Infrared ( FT-IR) Spectrometry Analysis

**Figure 4:**
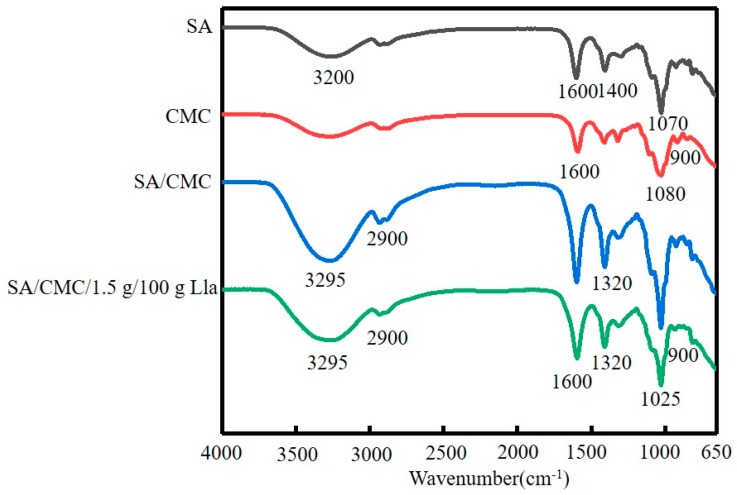
FT-IR spectra of SA, CMC, SA/CMC, SA/CMC/1.5 g/100 g Lla films.

FT-IR measurements were performed on pure SA films, pure CMC films, SA/CMC films, and SA/CMC/LAB films to analyze intermolecular interactions between sodium alginate and sodium carboxymethylcellulose and to analyze the effect of *Lactococcus lactis* addition on the intermolecular forces in the films. As shown in Figure 4, the -OH stretching peak around 3295 cm^−1^ was observed, originating from glucose residues in sodium alginate [34], which contains a large number of -OH groups [35]. Meanwhile, an absorption peak was observed near 1610 cm^−1^, indicating the presence of C=O stretching bonds in the edible films [36]. The absorption peak near 1400 cm^−1^ was attributed to the 3, 6 glycoside bridge in galactoside [37]. Given the presence of galactose in sodium alginate, a C-4 absorption peak appeared at 1070 cm^−1^ [38], while the absorption peak at 900 cm^−1^ was due to the C-O-C structure in galactose [39]. Based on the IR spectra, it was confirmed that the addition of *Lactococcus lactis* did not affect the chemical bonds in the edible films; hence, their excellent chemical properties were maintained. This also indirectly indicates that sodium alginate-sodium carboxymethylcellulose films can effectively incorporate *Lactococcus lactis*, verifying that this strategy is feasible.

### 2.8. Viability and Antimicrobial Activity of the Film

The viability of *Lactococcus lactis* in different films stored for 20 days at 4 °C was shown in Figure 4a. Due to that the films based on polysaccharide improved the viability of *Lactococcus lactis*, the initial population of *Lactococcus lactis* remained constant during 5 days (*p* > 0.05). After 10 days, a slight decrease was observed. There are several factors must be considered, such as water activity, temperature and presence of oxygen [22]. Dong et al. demonstrated that the structural and physical properties of the films can influence the viability of *Lactococcus lactis* [40]. the edible films with high amounts of solutes would increase molecular mobility, and it facilitates the occurrence of enzymatic and chemical reactions that damage essential cellular structures and phospholipid membrane bilayers [22]. On the 20th day, the 1.5 g/100 g film was the most effective ability to maintain the viability of *Lactococcus lactis*, and provided the highest protection allowing the retention of the 91.74% ± 2.36% of the initial number of living *Lactococcus lactis*. The 1.5 g/100 g film has the lowest WVP and moisture content, which have been reported as an efficient strategy for improving viability of *Lactococcus lactis* in food systems [14].

**Figure 5:**
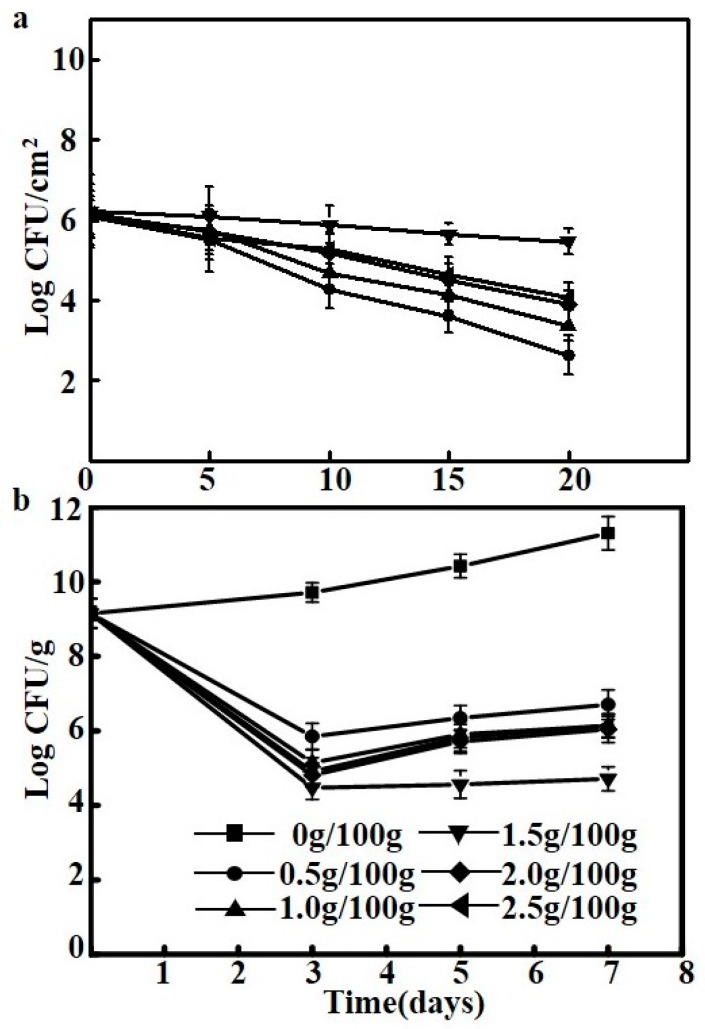
(**a**) The viability of *Lactococcus lactis* in different films for 20 day; (**b**) effect of bioactive films on the growth of *Staphylococcus aureus* on TSA medium stored at 4 °C.

The antibacterial activity of the *Lactococcus lactis*-containing films was tested on tryptic soy agar (TSA) culture medium by storing the medium in an incubator at 4 °C for one week, where film without *Lactococcus lactis* was used as control sample (Figure 5b). The experimental results, as shown in Figure 5b, revealed that bacteria-free films did not exhibit antibacterial activity, as expected, and *Staphylococcus aureus* growth was observed (viable cell counts of 9.15 ± 1.21 to 10.43 ± 2.07 log CFU/g at 4 °C). This may be attributed to these films providing some nutrients (in addition to those from the TSA culture media) for *Staphylococcus aureus* growth [14]. In contrast, all the films with added *Lactococcus lactis* showed significant antibacterial activity (*p* < 0.05). On the third day of storage, all *Lactococcus lactis*-containing films achieved the optimal antibacterial behavior, where the 1.5 g/100 g film showed the best antibacterial performance. This result is similar to that of Sánchez-González et al. who showed that the addition of bacteria to films resulted in the best inhibitory effect on the growth of *Staphylococcus aureus* [14]. Natrajan et al. found that nisin, a metabolic product of *Lactococcus lactis* in *Lactococcus lactis*-containing edible films, was bactericidal and diffused to the film surface where microbial proliferation was inhibited [41].

In addition, it was also found that the films with 2.0 g/100 g *Lactococcus lactis* showed a slightly lower bacteriostatic performance than those with 1.5 g/100 g *Lactococcus lactis*; this may be due to the latter having a larger quantity of viable bacterial cells than the former after drying. Moreover, the film with 1.5 g/100 g *Lactococcus lactis* appears to be more favorite environments for the viability of *Lactococcus lactis*. Previous studies reported a similar positive effect of sodium caseinate films on the survival of other lactic acid bacteria [13,14]. It was found that with increased culture time, the number of viable *Staphylococcus aureus* cells tended to increase, which was primarily due to some of the *Lactococcus lactis* in the films dying after long incubation times [15], leading to a decrease in the concentration of bacteriocin in the films. Higher initial viable cell contents in the films resulted in slower growth of *Staphylococcus aureus*. Over a seven-day bacteriostatic test period, all *Lactococcus lactis*-containing films showed good antibacterial activity; by the seventh day, the 1.5 g/100 g films showed a decrease in the viable cell counts of *Staphylococcus aureus* by four logarithmic units.

## 3. Materials and Methods

### 3.1. Materials

Sodium alginate (viscosity ≤ 0.02 Pa*s for an aqueous solution of 1 wt% at 20 °C), sodium carboxymethylcellulose, and glycerol were purchased from Sigma-Aldrich (Sigma chemicals, St. Louis, MO, USA). Stock cultures of *Lactococcus lactis* ATCC 11454 and *Staphylococcus aureus* ATCC 6538 was kept frozen (−80 °C) in synthetic media enriched with 30% glycerol.

### 3.2. Preparation of the Bioactive Films

Given that the activity of *Lactococcus lactis* ATCC 11454 decreases during storage at 4 °C, it is necessary to activate the bacteria before testing. Two inoculation loops of *Lactococcus lactis* culture preserved in paraffin were inoculated into liquid MRS (pH = 6.4 ± 0.3) culture medium that had first been subject to steam sterilization in an autoclave at 121 °C for 15 min and then cooled to room temperature, where the bacteria were subject to 2–3 incubation cycles at 25 °C to achieve the required viability, after which the culture was centrifuged at 3000 rpm for 10 min and then rinsed three times with saline that had been sterilized at 121 °C for 15 min to obtain the bacteria for later use [42].

Aqueous solutions for making films were prepared (*w*/*w* or *v*/*w* for solute/solvent) containing 2 g/100 g of sodium alginate, 0.3 g/100 g of sodium carboxymethylcellulose, and 1 mL/100 g of glycerol. The bacteria collected by centrifugation were then added to the solution (pH = 6.5 ± 0.3) at a level of 0, 0.5, 1.0, 1.5, 2.0, and 2.5 g/100 g of film solution. The resulting mixture-hydrosol was stirred in a 30 °C water bath for 30 min to ensure complete mixing, then the mixture-hydrosol was allowed to settle in the water bath for 30 min to remove any foam. Finally, 25 g of the prepared *Lactococcus lactis*-containing mixture was poured on to a glass plate and allowed to dry at 30 °C for 48 h. After film formation, the film was removed and placed in a climate-controlled chamber (50% relative humidity and 25 °C) for 24 h to ensure equilibrium for later use.

### 3.3. Determination of pH

As one of the important factors affecting the viability of *Lactococcus lactis*, it is necessary to measure the pH. Appropriate amounts of liquid MRS culture medium, mixture-hydrosol, edible film, and Na_2_HPO_4_ (10^−3^ M Na_2_HPO_4_ served as buffer in all experiments) were measured and put into a beaker. An acidity meter (Chengdu century ark technology Co., Ltd., Chengdu, China) was used to measure the pH. In this study, we adapted the method, where 1 g of the *Lactococcus lactis*-containing edible films was added to 9 mL of neutral water and stirred for 30 min to ensure the pH of the dry film. For each *Lactococcus lactis* content, three similar film samples were measured six times.

### 3.4. Enumeration of Lactococcus lactis

Given that the maintenance of bioactivity of the *Lactococcus lactis* in edible films is critical for the bacteriostatic preservation effect [15], it is necessary to measure the viable cell counts in *Lactococcus lactis*-containing films. In this study, we adapted the method of López De Lacey et al. [43], where 1 g of the *Lactococcus lactis*-containing edible films was added to 9 mL of sterile saline under constant stirring to ensure complete dissolution. The resulting solution was then sequentially diluted multiple times with sterile saline. For each dilution, portions of the diluted solution were coated on MRS agar in culture dishes, which were then placed in a bacterial incubator for anaerobic incubation at 25 °C for 48 h to allow growth of the colonies, followed by bacterial counting of the MRS agar plates, where the total bacterial count is expressed as log colony-forming units per gram (log CFU/g) [44].

### 3.5. Mechanical and Optical Properties

An electronic tensile tester (Shenzhen Sans Test Machine Co., Ltd., Shenzhen, China) was used to characterize the mechanical properties. Before the measurement, the edible films were equilibrated under a relative humidity of 50% for 24 h, and the films were cut into 60 mm × 10 mm strips, which were then immobilized using the testing jigs. We used a jig distance of 40 mm and a stretching speed of 100 mm/min to measure the tensile strength and elongation of the edible films. For each *Lactococcus lactis* content, three similar film samples were measured six times. The TS (MPa) was calculated according to the following formula:

(1) $$\mathrm{TS}=\frac{F}{L\times W}$$

where F is the maximum tensile force (N) when the film breaks, L is the average thickness of the film (mm), and W is the width of the sample (mm). The E was calculated according to the following formula:

(2) $$E(\%)=\frac{L_{1}-L_{0}}{L_{0}}\times 100$$

where E is the elongation (%), L_0_ is the length of a film sample before testing, and L_1_ is the length of the sample when it breaks.

For each *Lactococcus lactis* content in the sodium alginate-sodium carboxymethylcellulose edible films, three parallel samples were used for film thickness measurements, in which the thickness of each film was determined using a thickness meter (Mitutoyo Manufacturing Co., Ltd., Tokyo, Japan) to measure the thickness (mm) at eight points on the film, which were averaged [45].

The color characteristics of the edible films were measured with a color difference meter (Konica Minolta Sensing, Inc., Osaka, Japan) using three parallel samples for each condition, which were measured in triplicate [27]. Take a standard plate as the control film (L* = 8 8.45, a* = 0.60, b* = 0.90). The total color difference of the synthetic biofilm was calculated according to the following formula

(3) $$\Delta E=\sqrt{{\left(\Delta L^{*}\right)}^{2}+{\left(\Delta a^{*}\right)}^{2}+{\left(\Delta b^{*}\right)}^{2}}$$

### 3.6. Water Vapor Permeability

The wet cup method was adapted in this study. Before the measurement, the films were equilibrated in a relative humidity of 75% for 2 h. In a moisture permeable cup, allochroic silica gel was added up to around 5 mm from the cup mouth, then the mouth was sealed with an edible film and the total mass was measured. Then, the cup was placed in an artificial climate chamber (75% relative humidity at 25 °C) to equilibrate. Weight measurements were performed every 8 h for three consecutive days, with three parallel samples for each type of edible film. The WVP was calculated according to the following formula:

(4) $$\mathrm{WVP}=\frac{m\times L}{A\times t\times \Delta P}$$

where, m is the mass (g) of water that permeates through the film; L is the thickness (m) of the film; A is the film area (m^2^) through which the water permeates; t is the water permeation time (s); ΔP is the water vapor pressure difference (Pa) across the film.

### 3.7. Moisture Content

The MC of the edible films was measured by slightly modifying the method presented by Kurek et al. [46]. Each film was cut into 2 cm × 2 cm specimens, which were weighed and then placed in an oven at 105 °C for 24 h. The MC values were determined using the following equation after measuring the weight loss of three parallel samples:

(5) $$\mathrm{MC}=\frac{w_{i}-w_{f}}{w_{i}}\times 100$$

where w_i_ is the mass of the initial sample and w_f_ is the mass of the dried sample.

### 3.8. Surface Morphology and FT-IR Analysis

Before the measurements, the films were dried and samples with a size of 2 mm × 5 mm were cut and fixed horizontally to the sample stage. The sample surfaces were gold sputtered using ion-plating equipment for 20–30 min under vacuum to produce a layer with a thickness of ~10 μm. Then, the samples were placed inside a SEM (FEI Quanta 200, Eindhoven, The Netherlands) and vacuum was applied for 20 min. The accelerating voltage of the electron beam was set to 15 kV. The surface structure of the films was observed. In addition, Fourier transform infrared (FT-IR, Bruker Gmbh, Ettlingen, Germany) spectra of the thin-film samples were measured over the wavenumber range of 4000–650 cm^−1^ using an FT-IR spectrometer.

### 3.9. Viability and Antimicrobial Properties of the Films

The viability of *Lactococcus lactis* in different films at 4 °C for 20 days. Briefly, the films were stored in Petri Dishes inside of desiccators and 75% relative humidity and every 5 days they were removed from the Petri dishes and placed in a sterile plastic bag with 100 mL of tryptone phosphate water. The bag was homogenized for 2 min in a Stomacher blender. Serial dilutions were made and then poured onto MRS agar. Plates were incubated for 48 h at 37 °C before colonies were counted [12].

Kristo’s method was adapted in this study to measure the antibacterial properties of the edible films containing different levels of *Lactococcus lactis* [47]. We added 20 g of TSA culture medium to culture dishes; then, the surface was inoculated with *Staphylococcus aureus* solutions that had been diluted to the desired concentration. Subsequently, the *Staphylococcus aureus* was covered by films containing different concentrations of *Lactococcus lactis*; all film samples had the same size. Next, the culture medium was placed in a constant-temperature incubator at 4 °C for incubation, during which bacterial counting of the *Staphylococcus aureus* on the TSA culture medium was conducted at regular intervals. Next, the culture medium was removed from the culture dishes and placed in a sterile bag, into which 80 mL of sterile saline was added, followed by homogenization using a homogenizer for 2 min. After this, 1 mL of the homogenized solution was transferred to 9 mL of sterile saline under constant stirring. The resulting mixture was serially diluted multiple times; each time, portions of the diluted mixture were first coated on the TSA agar and then placed in an incubator at 37 °C for 48 h, followed by bacterial counting. All tests were performed in triplicate.

### 3.10. Statistical Analysis

The test data are expressed as mean ± standard deviation (SD). Analysis of variance and significance testing were performed using Duncan in statistical analysis software SPSS 22, and mapping was performed with Origin 2017.

## 4. Conclusions

The addition of *Lactococcus lactis* did not significantly change the thickness of the films, but changed the color, thickness, and luster of the films and enriched the composition, resulting in the films undergoing significant changes in the physical properties, where both the tensile strength and elongation at break decreasing significantly, while the water vapor permeability significantly increased. Although the viability of *Lactococcus lactis* decreased slightly during film drying, *Lactococcus lactis*-containing films still had a significant antibacterial effect on *Staphylococcus aureus* for up to one week in a low-temperature environment, where the effect was the most obvious for the 1.5 g/100 g films. The effectiveness of the bacteriostatic effect of the films depends on the amount of added *Lactococcus lactis*, the types of foods to be packaged, as well as the types and initial amounts of harmful bacteria. This study, amongst others on *Lactococcus lactis*-containing films, provides a new method and strategy for antibacterial preservation of foods.

## Figures and Tables

**Table 1:** Effect of the incorporation *Lactococcus lactis* on thickness, water vapor permeability (WVP), and mechanical properties.

Edible Film	Thickness (mm)	WVP (10^−11^ g m/m^2^ s Pa)	TS (MPa)	E (%)
SA/CMC/0 g/100 g Lla	0.034 ± 0.02 ^a^	6.73 ± 0.68 ^cd^	27.23 ± 0.09 ^a^	24.86 ± 1.65 ^a^
SA/CMC/0.5 g/100 g Lla	0.034 ± 0.01 ^a^	6.09 ± 0.81 ^d^	24.42 ± 3.03 ^b^	24.17 ± 1.61 ^ab^
SA/CMC/1.0 g/100 g Lla	0.039 ± 0.02 ^a^	7.14 ± 0.91 ^c^	19.04 ± 2.66 ^c^	22.33 ± 1.51 ^bc^
SA/CMC/1.5 g/100 g Lla	0.041 ± 0.01 ^a^	8.60 ± 1.05 ^b^	16.89 ± 2.59 ^d^	21.13 ± 1.43 ^c^
SA/CMC/2.0 g/100 g Lla	0.041 ± 0.02 ^a^	9.31 ± 1.17 ^b^	9.34 ± 1.14 ^e^	14.18 ± 1.21 ^d^
SA/CMC/2.5 g/100 g Lla	0.043 ± 0.01 ^a^	10.25 ± 1.25 ^a^	7.50 ± 1.01 ^f^	9.19 ± 1.03 ^e^

^a–f^ Different letter between rows indicate significantly different values (*p* < 0.05) according to Duncan’s post hoc means comparison test. Data are presented as mean ± SD (*n* = 3).

**Table 2:** Effect of the incorporation of *Lactococcus lactis* on color characteristics of biopolymer films.

Edible Film	L*	a*	b*	△E
control	88.45 ± 1.82 ^c^	0.60 ± 0.64 ^e^	0.90 ± 0.77 ^f^	
SA/CMC/0 g/100 g Lla	89.81 ± 1.81 ^a^	1.14 ± 0.77 ^d^	3.52 ± 0.35 ^e^	3.00 ± 0.45 ^c^
SA/CMC/0.5 g/100 g Lla	89.24 ± 1.63 ^b^	1.36 ± 0.87 ^c^	5.28 ± 0.67 ^c^	4.45 ± 0.58 ^b^
SA/CMC/1.0 g/100 g Lla	88.94 ± 1.57 ^b^	1.24 ± 0.81 ^d^	4.75 ± 0.54^d^	4.00 ± 0.53 ^bc^
SA/CMC/1.5 g/100 g Lla	87.82 ± 1.43 ^d^	1.74 ± 0.14 ^b^	5.85 ± 0.79 ^b^	5.10 ± 0.66 ^b^
SA/CMC/2.0 g/100 g Lla	87.76 ± 1.41 ^d^	1.78 ± 0.15 ^ab^	5.84 ± 0.78 ^b^	5.09 ± 0.65 ^b^
SA/CMC/2.5 g/100 g Lla	87.06 ± 1.28 ^e^	1.86 ± 0.18 ^a^	7.69 ± 0.95 ^a^	7.05 ± 0.86 ^a^

^a–f^ Different letter between rows indicate significantly different values (*p* < 0.05) according to Duncan’s post hoc means comparison test. Data are presented as mean ± SD (*n* = 3). SA, sodium alginate; CMC, sodium carboxymethylcellulose.
